# KnockTF: a comprehensive human gene expression profile database with knockdown/knockout of transcription factors

**DOI:** 10.1093/nar/gkz881

**Published:** 2019-10-10

**Authors:** Chenchen Feng, Chao Song, Yuejuan Liu, Fengcui Qian, Yu Gao, Ziyu Ning, Qiuyu Wang, Yong Jiang, Yanyu Li, Meng Li, Jiaxin Chen, Jian Zhang, Chunquan Li

**Affiliations:** 1 School of Medical Informatics, Daqing Campus, Harbin Medical University, Daqing 163319, China; 2 Department of Pharmacology, Daqing Campus, Harbin Medical University, Daqing 163319, China

## Abstract

Transcription factors (TFs) and their target genes have important functions in human diseases and biological processes. Gene expression profile analysis before and after knockdown or knockout is one of the most important strategies for obtaining target genes of TFs and exploring TF functions. Human gene expression profile datasets with TF knockdown and knockout are accumulating rapidly. Based on the urgent need to comprehensively and effectively collect and process these data, we developed KnockTF (http://www.licpathway.net/KnockTF/index.html), a comprehensive human gene expression profile database of TF knockdown and knockout. KnockTF provides a number of resources for human gene expression profile datasets associated with TF knockdown and knockout and annotates TFs and their target genes in a tissue/cell type-specific manner. The current version of KnockTF has 570 manually curated RNA-seq and microarray datasets associated with 308 TFs disrupted by different knockdown and knockout techniques and across multiple tissue/cell types. KnockTF collects upstream pathway information of TFs and functional annotation results of downstream target genes. It provides details about TFs binding to promoters, super-enhancers and typical enhancers of target genes. KnockTF constructs a TF-differentially expressed gene network and performs network analyses for genes of interest. KnockTF will help elucidate TF-related functions and potential biological effects.

## INTRODUCTION

Transcription factors (TFs) can activate or repress expression of genes that are proximal or distal to their DNA binding sites (1). A lot of studies have shown transcriptional control of TFs by binding to promoters or enhancers of downstream target genes (2,3). TFs and their target genes are important in human diseases and biological processes (4). Upstream signaling pathways further regulate TFs and alter the expression levels of downstream target genes (5). With the emergence of high-throughput techniques, Chromatin immunoprecipitation coupled with next-generation sequencing (ChIP-seq) technique and gene expression profile analysis technique before and after knockdown or knockout have become the two most important strategies for obtaining target genes of TFs and exploring TF functions. For example, ChIP-seq was used to identify STAT1 targets in human HeLa cells (6) and MyoD binding sites in skeletal muscle cells (7). ChIP-seq based on direct ultrahigh-throughput DNA sequencing was used to map *in vivo* binding of the neuron-restrictive silencer factor REST to its locations in the human genome (8). The locations of the sequence-specific TFs Nanog, Oct4, STAT3, Smad1, Sox2, Zfx, c-Myc, n-Myc, Klf4, Esrrb, Tcfcp2l1, E2f1 and CTCF and transcription regulators p300 and Suz12 were generated using high-throughput ChIP-seq datasets, which were known to play different roles in embryonic stem cell biology (9). To systematically determine the target genes of TFs, the Encyclopedia of DNA Elements (ENCODE) consortium generated 424 ChIP-seq profiles including >120 human TFs from various cell lines (10). A large number of studies show that gene expression profile analysis before and after knockdown or knockout effectively helps identify target genes of TFs and explore TF functions. Examples are 269 TF knockout microarrays used for genome-scale investigation of eukaryotic gene regulation (11), *Gata1* knockout to identify GATA1-responsive genes (12), and tumor cell-specific *Twist1* knockout to study the effect of Twist1 on breast tumors *in vivo* (13). More than 200 gene expression profiles for TF knockdown or knockout are provided by ENCODE, involving 145 human TFs from four cell lines (14). These studies demonstrate the importance and widespread utility of TF ChIP-seq and knockdown/knockout techniques for addressing key issues associated with cancer biology and disease development.

Numerous databases have ChIP-seq as a central method for mapping and analyzing TFs and their binding sites at genome-wide scale, such as GTRD (15), DPRP (16), dbCoRC (17), Cistrome Cancer (18), ENCODE (14), ReMap (19), ChIP-Atlas (20) and Factorbook (21). These TF ChIP-seq databases provide valuable data and effective platforms for deciphering the mechanisms of transcriptional regulation. However, up to now, gene expression profile databases of TF knockdown and knockout, as another type of the important strategy for obtaining target genes of TFs and exploring TF functions, are still not built. With the development of studies on human diseases and biological processes, TF knockdown and knockout data are accumulating rapidly. Human gene expression profile datasets of TF knockdown and knockout create an urgent need to comprehensively and effectively collect and process these data. More importantly, a large number of studies show that upstream pathways and downstream target genes of TFs are strongly associated with TF biological functions (22). In addition, information about TF binding to promoter, super-enhancer (SE) and typical enhancer (TE) regions of target genes is crucial (23). Therefore, detailed information on TFs such as their upstream pathways, downstream target genes, and binding to promoters, SEs and TEs of genes should be provided for explaining and analyzing the regulation mechanism of TFs.

Motivated by the lack of available resources, we developed a comprehensive human gene expression profile database of TF knockdown and knockout. KnockTF (http://www.licpathway.net/KnockTF/index.html) provides a large number of available resources for human gene expression profile datasets associated with TF knockdown and knockout and annotates TFs and their target genes in a tissue/cell type-specific manner. The current version of KnockTF has 570 manually curated RNA-seq and microarray datasets associated with 308 TFs disrupted by different knockdown and knockout techniques and across different tissue and cell types. KnockTF provides comprehensive gene expression information about target genes of TFs of interest and collects upstream pathway information of TFs and various functional annotation and analysis results of downstream target genes, including Gene Set Enrichment Analysis, Gene ontology enrichment, KEGG pathway enrichment, hierarchical clustering analysis and differentially expressed analysis. KnockTF also provides detailed information about TFs binding to promoters, SEs and TEs of target genes. In addition, a TF-differentially expressed gene network is constructed and used to perform network analyses for gene sets of interest. KnockTF provides a conveniently, user-friendly interface for querying, browsing, analyzing and downloading detailed information about human gene expression profile datasets of TF knockdown and knockout. KnockTF will be helpful for elucidating TF-related functions and exploring potential biological mechanisms.

## DATA SOURCE AND PROCESSING

### Collection and treatment of TF knockdown/knockout datasets

**Figure 1.**
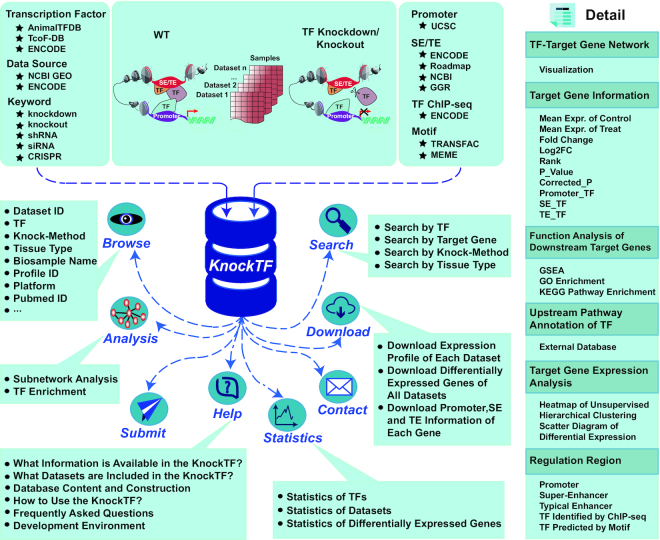
Database content and construction. KnockTF has a large number of manually curated human gene expression profile datasets of TF knockdown and knockout and a user-friendly interface to query, browse, analyze and download detailed information about these datasets.

A list of >1300 TFs was collected from AnimalTFDB (24), TcoF-DB (25) and ENCODE (14) (Figure 1). We manually assigned two generic-level classifications (superclass and class) to TFs according to TFClass, which classifies eukaryotic TFs according to DNA-binding domains (26). TFs that were not classified by TFClass were further classified according to TcoF-DB (25) and ENCODE (14). We searched NCBI GEO (27) and ENCODE (14) databases to retrieve TF knockdown and knockout data using a list of keywords, such as ‘knockdown’, ‘knockout’, ‘shRNA’, ‘siRNA’ and ‘CRISPR’. Data were manually checked to ensure high quality. Preliminary screening results were further traversed in title, summary and protocol for samples to classify TF datasets with different knockdown or knockout techniques and across different tissue or cell types. As a result, 364 datasets were collected from the NCBI GEO database, for 185 TFs from 266 series and 51 platforms. From the ENCODE database, 206 datasets were collected. These datasets involved six knockdown and knockout techniques, including shRNA (28), siRNA (29), esiRNA (30), CRISPRko (31), CRISPRi (32) and CRISPRedit (33). Thus, we manually curated 570 RNA-seq and microarray datasets associated with 308 TFs disrupted by six knockdown and knockout techniques and across multiple tissue and cell types (Supplemental Table S1).

We downloaded all the gene expression profiles corresponding to the 570 datasets from GEO and ENCODE. For each GEO expression profile, probes were mapped to gene symbols. Probes mapped to multiple gene symbols were deleted and multiple probes mapped to the same gene symbol were merged by averaging expression values. From ENCODE, we downloaded gene quantification files for knockdown/knockout and control samples, and merged them for gene expression profiles. For each gene expression profile, Ensembl IDs were mapped to gene symbols. Genes with zero values in all knockdown/knockout or control samples were deleted. Log_2_ transformation was performed for gene expression profiles with raw expression values and fold change (FC) was computed for each gene. Statistical significance for differential expression was computed for gene expression profiles in datasets with the number of samples ≥3 by limma, a common, effective R/Bioconductor software package for differential expression analyses (34).

### Analysis of TF knockdown/knockout datasets

In KnockTF, we annotated upstream pathways of TFs and conducted functional annotations and analyses of downstream target genes before and after TF knockdown or knockout, including Gene Set Enrichment Analysis (GSEA) (35), Gene ontology (GO) enrichment (36,37), KEGG pathway enrichment (38), hierarchical clustering analysis and differentially expressed analysis. First, genes in each TF knockdown or knockout dataset were ordered in a ranked list based on FC values. GSEA was used to determine if genes from particular pathways were statistically significant for different phenotypes (35). Using the results, KnockTF listed the top 20 up-regulated pathways and the top 20 down-regulated pathways with enrichment score, normalized enrichment score, nominal P-value and FDR. Second, GO enrichment and KEGG pathway enrichment analyses of the top 100, 200, 300, 400 and 500 downstream target genes ranked by FC values were determined by hypergeometric test. KnockTF displayed enriched GO terms, KEGG pathways and corresponding –log10 P-values. Then, the top 100 up-regulated and down-regulated genes for each TF knockdown/knockout dataset were used for unsupervised hierarchical clustering. Gene expression profiles were shown as heatmaps with corresponding dendrograms. Finally, KnockTF performed differentially expressed analyses under the threshold of FC ≥3/2 & FC ≤2/3 and showed up-regulated and down-regulated genes and other genes as scatter diagrams with FC values.

### TF-differentially expressed gene network

KnockTF constructed a TF-differentially expressed gene (DEG) network. First, for each TF knockdown or knockout dataset, we extracted DEGs under the threshold of FC ≥3/2 & FC ≤2/3 and formed TF-DEG pairs that were ranked based on significant levels of DEGs. Second, we combined all TF-DEG pairs for the 570 TF knockdown and knockout datasets. If a TF-DEG pair appeared multiple times in different TF knockdown or knockout datasets, we removed duplications and retained its minimum rank. Then, we reordered all nonredundant TF-DEG pairs and constructed a TF-DEG network with TFs and their DEGs as nodes and TF-DEG pairs as edges. The rank of TF-DEG pairs represented the importance of the regulatory intensity of TFs on target genes. TF-target relationships supported by the ChIP-seq data were also marked and recorded for TF-DEG pairs. Topological features such as degree, betweenness and closeness of all nodes in the TF-DEG network were computed. By mapping genes of interest to the TF-DEG network, KnockTF located a subnetwork and computed topological features of subnetwork genes. The subnetwork consisted of genes of interest and their one-step neighbors within the TF-DEG network. TF-target gene relationships supported by the ChIP-seq data were represented as bold edges in the subnetwork. The size of the subnetwork could be adjusted by filtering the number of the most important TF-DEG pairs. KnockTF can also compute hypergeometric test between genes of interest and DEGs regulated by each TF to obtain the most important TFs regulating the genes.

### Annotation of TF binding regions of target genes

KnockTF defines the promoter region of a gene as a basal domain of −2 kb to +2 kb around the transcription start site. More than 330 000 SE regions and 6 500 000 TE regions involving 542 tissue/cell types were obtained from SEdb, which was previously developed by our group (39). KnockTF mapped SE and TE regions for every gene using four linking strategies: closest active genes, overlapping genes, proximal genes and closest genes (40,41).

To identify TFs binding to promoters, SEs, and TEs of genes, we collected 1137 TF ChIP-seq datasets from ENCODE containing 457 TFs and >25 000 000 TF binding sites in 106 cell or tissue types (Supplemental Table S2). TF binding peaks overlapping with the promoter, SE or TE regions of genes in all TF knockdown/knockout datasets were identified using BEDTools (v2.25.0) (42). To identify TF motifs in promoter, SE or TE regions, >3000 DNA binding motifs for ∼700 TFs were collected from the TRANSFAC (43) and MEME (Multiple Em for Motif Elicitation) suite (44). Motif occurrences within the promoter, SE or TE regions of genes were identified using FIMO (Find Individual Motif Occurrences) with a threshold of *P*< 1e–5 (45).

## DATABASE USE AND ACCESS

### Search interface for conveniently retrieving TF knockdown/knockout datasets

**Figure 2.**
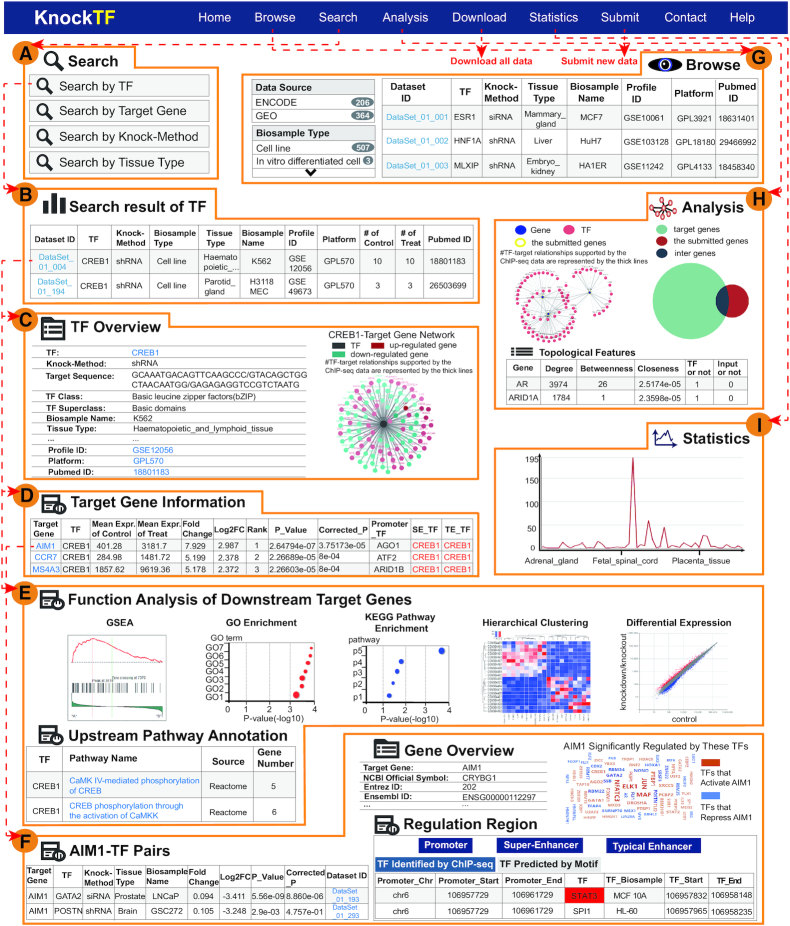
Main functions and usage of KnockTF. (**A**) Four inquiry modes are available. (**B**) Table of search results including Dataset ID, TF, Knock-Method, Biosample Type, Tissue Type, Biosample Name, Profile ID, Platform and Pubmed ID. (**C**) Overview of TF knockdown/knockout dataset. (**D**) Detailed interactive table of target gene information. (**E**) Details about upstream pathways of the TF of interest and various functional annotations and analyses of downstream target genes. (**F**) Interactive table with detailed descriptions about genes of interest. (**G**) Browsing details of TF knockdown/knockout datasets. (**H**) Online tool for TF-target gene network analysis. (**I**) Quantitative statistics for TF knockdown/knockout datasets.

KnockTF provides four kinds of inquiry modes: ‘Search by TF’, ‘Search by Target Gene’, ‘Search by Knock-Method’ and ‘Search by Tissue Type’ (Figure 2A). In TF-based inquiry mode, users input a TF of interest or select a TF according to TF class or superclass of interest. Clicking ‘Search’ gives users TF knockdown or knockout datasets associated with the TF. In target gene-based inquiry mode, users input a gene using Gene Symbol, Entrez ID or Ensembl ID, then use FC ≥2 & FC ≤1/2 or FC ≥3/2 & FC ≤2/3 to filter TF knockdown or knockout datasets in which the input gene is significantly expressed. In knock-method-based inquiry mode, users query related TF knockdown or knockout datasets by selecting a knock-method, data source and biosample type. In tissue type-based inquiry mode, users input a tissue type, biosample name, biosample type and data source to query the related TF knockdown or knockout datasets.

Brief search results are presented as a table in the result page (Figure 2B). Users click ‘Dataset ID’ to view details about TF knockdown or knockout datasets, such as TF overview, TF-target gene network and target gene information before and after TF knockdown or knockout (Figure 2C). In the table of target gene information, an interactive table describes target gene; TF; mean expression of control samples; mean expression of knockdown/knockout samples; FC; log_2_FC; rank; *P*-value computed by limma; TFs binding to promoter, SEs, and TEs identified by ChIP-seq/motif and the number of these TFs (Figure 2D). In addition, KnockTF lists more detailed information about upstream pathway information of TFs and various functional annotation and analysis results of downstream target genes, including GSEA, GO enrichment, KEGG pathway enrichment, hierarchical clustering analysis and differentially expressed analysis (Figure 2E). Detailed descriptions for each gene are shown on new page after clicking ‘Target Gene’ in the table of target gene information. Descriptions include gene overview, differentially expressed target gene (FC≥3/2 & FC≤2/3)-TF pairs and annotation of TF binding regions of target genes of interest (Figure 2F). KnockTF also provides gene expression atlas from different sources, such as GTEx (46), CCLE (47), TCGA (https://cancergenome.nih.gov/) and ENCODE (14).

### User-friendly interface for browsing TF knockdown/knockout datasets

The ‘Browse’ page is organized as an interactive table for quickly searching for TF knockdown or knockout datasets and customizing filters using ‘Data Source’, ‘Biosample Type’, ‘Tissue Type’, ‘TF Superclass’ and ‘TF’. Users can click ‘Show entries’ in a dropdown menu to change the number of records displayed per page. To view details of a TF knockdown or knockout dataset, users click on ‘Dataset ID’ (Figure 2G).

### Effective online tool for TF–target gene network analysis

To interactively analyze and view TF–target gene interactions, KnockTF constructs a TF–DEG network and provides network analysis tools, including subnetwork location, topological analysis and hypergeometric enrichment (Figure 2H). Using the ‘Subpathway Analysis’ tool, users submit a gene list to locate a subnetwork. The subnetwork consists of submitted genes and their one-step neighbors within TF–DEG network. TF–target gene relationships supported by the ChIP-seq data have bold edges in the subnetwork. Users can choose subnetwork size displays by filtering the number of the most important TF–DEG pairs. KnockTF also provides topological features of subnetwork genes including degree, betweenness and closeness. Using the ‘TF Enrichment’ tool, users can submit a gene list and set (FDR-adjusted) *P*-value for TF enrichment. KnockTF maps submitted genes to the TF–DEG network and performs hypergeometric test between submitted genes and all DEGs regulated by each TF. A result table lists TFs, intersection genes, the number of intersection genes and *P*-values for hypergeometric test. These TFs are under the threshold of (FDR-adjusted) *P*-value user sets that are considered the most important TFs that significantly regulate the submitted genes. KnockTF also provides the results of hypergeometric enrichment as Venn diagrams.

### Data download and statistics

KnockTF allows data downloading in ‘.txt’ format, mainly including gene expression profile of each dataset; differential expression information of genes; the promoter, SE, and TE regions; and TF binding information of target genes. In addition, KnockTF supports export of query results for each search result page. In the ‘Statistics’ page, KnockTF provides statistics on TFs by data source and statistics of datasets by knock-method and biosample type (Figure 2I). The number of DEGs (FC ≥ 3/2 & FC ≤ 2/3) for each TF knockdown or knockout dataset is also provided.

### Data submission

KnockTF encourages sharing TF knockdown/knockout data. We recommend that users submit TF, knock-method and biosample name, as well as a link to their data source. To ensure data quality, we check the submitted data before updating. Finally, we update the database dynamically based on the number of new datasets to ensure timely data release.

## DISCUSSION

The field of TFs is progressing fast and is one of the most investigated research areas (48). Identification of TFs and their target genes is pivotal for understanding the mechanisms of disease development and biological processes (4). Human gene expression profile datasets of TF knockdown and knockout are accumulating rapidly. These datasets are informative for obtaining target genes of TFs and elucidating TF biological functions. Based on the urgent need to comprehensively collect and process these data, we developed KnockTF, the first human gene expression profile database of TF knockdown and knockout with the largest number of TF knockdown and knockout expression data and the most comprehensive annotation information. KnockTF has 570 manually curated RNA-seq and microarray datasets associated with 308 TFs disrupted by different knockdown/knockout techniques and across different tissue/cell types. It provides a convenient database platform for exploring expression information of TFs and their regulated genes. As two most important TF research strategies, ChIP-seq and knockdown/knockout methods provide complementary analysis of TFs. Compared to existing TF databases that are based on data mainly from ChIP-seq, KnockTF effectively collects human gene expression profile data of TF knockdown and knockout. Thus, KnockTF is a new type of TF database that complements existing TF databases with its interest in TF knockdown/knockout data.

We established this database prompted by a great need of cell/molecular biologists, geneticists and data scientists to understand TF functions. Researchers can focus on genes that are differentially expressed before or after TF knockdown or knockout and further explore underlying mechanisms and biological functions. KnockTF mainly provides the following information to show our advantages: (I) differential expression analysis of genes before and after knockdown or knockout of TFs of interest; (II) TF-target gene network for visually displaying a TF of interest and target genes with the most up-regulated/down-regulated/differential expression; (III) important up-regulated and down-regulated pathways associated with genes before and after knockdown or knockout of TFs of interest; (IV) enriched GO terms and KEGG pathways; (V) upstream pathway annotation of TFs of interest; (VI) heatmaps from unsupervised hierarchical clustering; (VII) scatter diagram of DEGs; and (VIII) detailed information about TFs binding to promoters, SEs, and TEs of target genes. Furthermore, KnockTF has an online network analysis tool to help users understand the relationship between genes of interest and TF knockdown/knockout data.

The current version of KnockTF stores the most abundant human gene expression profile datasets of TF knockdown and knockout. However, much TF knockdown and knockout data may be available about other species in other data sources. In the next version of KnockTF, we will manually curate more TF knockdown and knockout data with more species and we will enrich the kinds of species. We encourage users to share TF knockdown/knockout data referring to different species. KnockTF aims to explore potential regulatory functions of TFs at the transcriptional regulation and epigenetic modulation levels. Continuous efforts will be made to update useful data and improve the functionality of the KnockTF database. Overall, the goal of the KnockTF database is to be a valuable resource for the scientific community for using TF knockdown and knockout data and exploring gene expression and transcriptional regulation in human diseases and biological processes.

## Supplementary Material

gkz881_Supplemental_Files
